# Efficacy of Oral Acetaminophen and Intravenous Chlorpheniramine Maleate versus Placebo to Prevent Red Cell Transfusion Reactions in Children and Adolescent with Thalassemia: A Prospective, Randomized, Double-Blind Controlled Trial

**DOI:** 10.1155/2018/9492303

**Published:** 2018-10-01

**Authors:** Piya Rujkijyanont, Chalinee Monsereenusorn, Pimpat Manoonphol, Chanchai Traivaree

**Affiliations:** ^1^Division of Hematology-Oncology, Department of Pediatrics, Phramongkutklao Hospital and College of Medicine, Bangkok, Thailand; ^2^Department of Pediatrics, Thungsong Hospital, Nakhon Sri Thammarat, Thailand

## Abstract

**Background:**

Thalassemia is a common congenital hemolytic disorder. In severe cases, regular blood transfusion is essentially required. The role of premedications to prevent transfusion reactions is varied among institutions with no standard guideline.

**Objective:**

To prospectively compare the risk of transfusion reactions in thalassemia patients premedicated with acetaminophen and chlorpheniramine maleate (CPM) versus placebo prior to blood transfusion.

**Material and Method:**

A randomized, double-blinded, placebo-controlled transfusion reaction study of 147 eligible patients was analyzed. All administered red blood cell (RBC) products were leukoreduced blood products. Patients were monitored and followed for the development of transfusion reactions for 24 hours after RBC transfusion.

**Results:**

A total of 73 patients randomized to receive active drugs consisting of acetaminophen and CPM were compared to 74 patients receiving placebo. The overall incidences of febrile reaction and urticarial rash were 6.9% and 22% in the patients randomized to receive active drugs comparing with 9.5% and 35.2% in the patients receiving placebo with no significant differences between two groups. However, delayed development of urticarial rash at 4-24 hours after RBC transfusion was significantly higher in female and patients receiving placebo.

**Conclusion:**

Administration of premedications in thalassemia patients receiving RBC transfusion without a history of transfusion reactions does not decrease the overall risk of transfusion reactions. However, the use of CPM might be beneficial to prevent delayed urticarial rash in those patients especially in females (Thai Clinical Trial Registry (TCTR) study ID: 20140526001).

## 1. Introduction

Thalassemia is a group of congenital hemolytic anemia commonly found worldwide. The pathophysiology of anemia in thalassemia resulted from reduced or absent synthesis of the alpha- or beta-globin chains of hemoglobin molecule leading to abnormal hemoglobin production [1–3]. The disease can be classified according to the affected globin chains of hemoglobin into alpha thalassemia in which alpha-globin chain production is affected and beta thalassemia in which beta-globin chain production is affected [4]. The unique characteristics of thalassemia include chronic hemolytic anemia, hepatosplenomegaly, failure to thrive, and other complications with a wide range of clinical spectrum depending on the disease's severity. In severe cases, these complications can be fatal if the patients are not treated appropriately [4–6]. The clinical and hematological spectrum of thalassemia disease has been simply categorized according to transfusion requirement into transfusion dependent thalassemia (TDT) and nontransfusion dependent thalassemia (NTDT) [7–9].

Thailand is known as one of the endemic areas of thalassemia disease. The prevalence of thalassemia traits in Thai population was reported to be 20–30% for *α*-thalassemia, 3-9% for *β*- thalassemia, and 20-30% for Hb E [10, 11]. The diverse thalassemia genotypes found in this population results in different phenotypic characteristics and variation in transfusion requirements comparing to other diseases [12, 13]. Given the clinical severity of ineffective erythropoiesis in patients with thalassemia disease, red blood cell (RBC) transfusion is considered as the mainstay of treatment in order to prolonged overall survival in most of the patients [14, 15]; however, a risk of developing reactions from RBC transfusion is commonly reported and quite challenging since the reactions are difficult to predict in each individual patient [16, 17].

Acute transfusion reactions can be categorized into immune-mediated, involving antigen-antibody complex formation, or nonimmune-mediated reactions. Immune-mediated reactions can be further divided into hemolytic and nonhemolytic reactions consisting of febrile and allergic reactions. Febrile reaction occurs at a rate of 0.3 to 6 percent of RBC transfusions in which fever usually occurs during the first 4 hours of transfusion with or without chills. The risk of febrile reaction is reduced to 0.2 percent when patients receive prestorage leukoreduced blood products but increased to 2 percent for poststorage leukoreduction [18]. Contrarily, the prevalence of allergic reactions occurs at a rate of 0.4 to 3 percent of all transfusions and is not mitigated by leukoreductions. Signs and symptoms of allergic reactions usually appear within 2 hours of transfusions consisting of urticarial rash, facial edema, airway edema, and lower respiratory tract symptoms, which are responsive to antihistamines. However, the symptoms might turn to be more severe such as hypotension or anaphylaxis. Patients who have experienced febrile or allergic reactions are at increased risk of recurrence of reactions with subsequent transfusions although the overall likelihood of reactions remains low [19].

Acetaminophen and chlorpheniramine maleate (CPM) are commonly used as premedications to prevent transfusion reactions from prestorage leukoreduced, poststorage leukoreduced, or nonleukoreduced blood products in most of the developing countries with variable practice guidelines among different institutions even though the literatures support that idea that pretransfusion medication is required only when transfusion reactions occur [18, 20–23]. The previous study [24, 25] suggested that there is no medical benefit of administering pretransfusion medication and also questioned the cost benefits of the practice. However, most of the studies were conducted on adult population with no specific disease type especially certain diseases that require regular transfusion. The aim of this study was to evaluate the need for pretransfusion medication prior to RBC transfusion specifically in children and adolescent with thalassemia disease. The primary objective of this study was to prospectively compare the risk of febrile and allergic reactions in those thalassemia patients who received oral acetaminophen and intravenous (IV) CPM versus placebo prior to RBC transfusion.

## 2. Materials and Methods

### 2.1. Patient Selection

One hundred and fifty-three thalassemia patients receiving RBC transfusion and attending the Hematology Clinic at Department of Pediatrics, Phramongkutklao Hospital, Bangkok, Thailand, from November 1, 2014, to October 31, 2017, were enrolled in the study. The informed consent and assent form were written and obtained from all participants as well as their parents or legal guardians prior to the enrollment in the study. The study protocol was approved by the Institutional Review Board of Phramongkutklao Hospital and Phramongkutklao College of Medicine, Bangkok, Thailand, following the ethical principles of the Declaration of Helsinki of 1975 and its revision. Inclusion criteria in this study included all children and adolescents with thalassemia aged between 1 and 22 years who required RBC transfusion. Patients were excluded from the study if they developed fever with body temperature of greater than 38°C prior to RBC transfusion or received antiallergic drugs within 24 hours or antipyretic drugs within 4 hours prior to the transfusion. The additional exclusion criteria included patients with a known history of allergy to either oral acetaminophen or IV CPM or those who had documented history of febrile or allergic transfusion reactions.

### 2.2. Study Design and Clinical Assessment

This study was a randomized, double-blind, placebo-controlled trial. All patients were randomly assigned to receive either active drugs or placebo in a double-blinded fashion with equal probability using randomization (block of four) to ensure approximately equal accrual over time. The dose of oral acetaminophen (500 mg tablet) was given at 250 mg (1/2 tablet) for patients who weighed less than 25 kg and 500 mg (1 tablet) for patients who weighed more than 25 kg. The dose of IV CPM was given at 0.35–0.4 mg/kg/dose with a maximum dose of 10 mg. The dispensing pharmacist, an individual not otherwise involved in the study, was aware of treatment allocation and dispensed either placebo or active drugs. Study medications were given 15 minutes before the RBC transfusion. All transfusions were closely monitored per standard practice guideline by the thalassemia clinic service for 4 hours during RBC transfusion and additional 20 hours at home. All other caregivers, members of the study team, and patients were blinded throughout the study duration and data collection until the study was completed. Transfusion reactions were evaluated using medical record review by the same investigators during the first 4 hours then parents were given a follow-up phone call by the research coordinator in order to assess patient's clinical status. Demographic data including patient's specific characteristics, age, sex, type of thalassemia, type of RBC products, and reported development of transfusion reactions were collected. A febrile reaction was defined as a body temperature of greater than 38°C. Urticarial reactions were classified as hives with or without itching. Other reactions, such as hemolytic transfusion reactions, facial edema, airway edema, and lower respiratory tract symptoms, were documented as described in the medical record.

### 2.3. Statistical Analysis

This study was designed to accrue 140 patients, which would have provided 90 percent power for detecting a difference risk of a transfusion reaction of 0.27 (treatment relative to placebo) at the two-sided level of significance. Baseline values of selected variables were calculated as the mean, standard deviation, minimum, maximum, frequency, and percentage. Differences between the two treatment groups with respect to age, sex, type of thalassemia, and type of RBC products were analyzed using Independent sample t-test for continuous variables and Chi-square test for the categorical variables. Variables used in the binary logistic regression model included age, sex, type of thalassemia, and type of RBC products, in addition to a treatment indicator (active drugs or placebo). Statistical Package for the Social Science (SPSS) version 23 software (IBM, NY, USA) was used for data analysis and p values of <0.05 were considered statistically significant.

## 3. Results

### 3.1. Patients' Demographic Data

**Figure 1:**
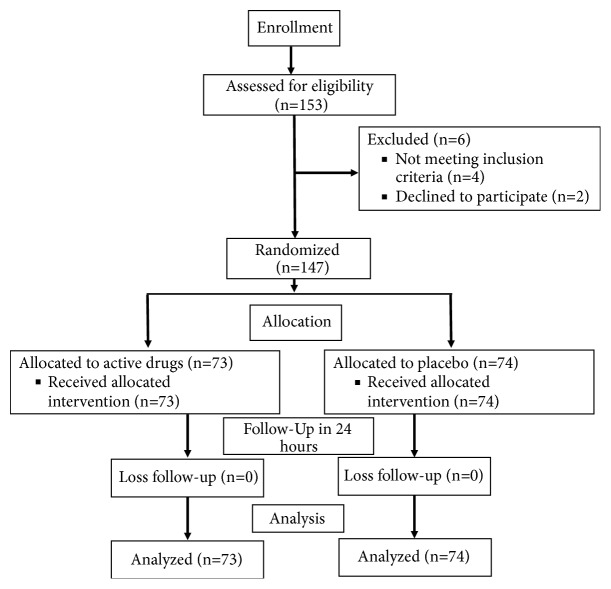
Study flow diagram.

As shown in Figure 1, one hundred and fifty-three patients were assessed for eligibility. Two patients refused to participate in the study and 4 patients were excluded due to a known history of allergy (2 patients) and taking antihistamine for upper respiratory tract symptoms (2 patients). One hundred and forty-seven patients met our inclusion criteria, and their parents or legal guardians provided informed consent to enroll in the study over 3 years beginning in November 2014. The patients were randomized according to the study protocol to receive active drugs (n=73) or placebo (n=74). The entire patients well complied with the study operation with no loss follow-up reported.

Patients' demographic data were shown in Table 1. The patients' age ranged between 1.50 and 21.17 years and all of them received RBC transfusion. Upon entering the study, the patients were randomized to receive either active drugs or placebo. Seventy-three patients were randomized to receive active drugs consisting of oral acetaminophen and IV CPM prior to RBC transfusion, and 74 patients were randomized to receive placebo. Of 73 patients randomized to receive active drugs, 37 patients (50.7%) were male and 36 (49.3%) were female. Among 74 patients receiving placebo, 45 patients (60.8%) were male and 29 (39.2%) were female. The mean ages of the patients randomized to receive active drugs and placebo were 12.49±4.84 and 11.32±4.72 years, and the mean ages at diagnosis of those patients were 24.93±23.84 and 25.08±24.36 months, respectively.

Beta thalassemia/hemoglobin E (ß thal/HbE) was the most common type of thalassemia disease in the study. The disease was noted in 99 patients in which 49 (67.1%) and 50 (67.5%) of those were randomized to receive active drugs and placebo, respectively. The second most common type of thalassemia disease was homozygous beta thalassemia (ß thal/ß thal), which was noted in 19 patients, and 12 (16.4%) and 7 (9.5%) of the patients received active drugs and placebo, respectively. AE Bart disease was noted in 16 patients, and 6 (8.2%) and 10 (13.5%) of those were randomized to receive active drugs and placebo, respectively. The rest of thalassemia type found in the study was Hemoglobin H disease with constant spring (Hb H with CS).

Among the different types of transfused RBC products, leukocyte poor packed red cell (LPRC) was transfused to 49 (67.1%) and 50 (67.6%) patients randomized to receive active drugs and placebo, respectively. In addition, leukocyte depleted packed red cell (LDPRC) was transfused to 24 (32.9%) patients randomized to receive active drugs and to the same numbers of patients who received placebo. There were no statistically significant differences in the patients' demographic data between 2 groups as described in Table 1.

### 3.2. The Incidence of Transfusion Reactions and Correlation with Premedications

 As shown in Table 2, the overall rate of febrile reaction during the first 24 hours after RBC transfusion was 6.9% in the patients randomized to receive active drugs comparing with 9.5% in the patients receiving placebo as pretransfusion medications (p value = 0.565). A febrile reaction within 4 hours of RBC transfusion was noted in 4 (5.5%) and 6 (8.1%) patients randomized to receive active drugs and placebo, respectively (p value = 0.527). Fever at 4 to 24 hours post RBC transfusion was reported in 1 patient (1.4%) randomized to receive active drugs and in 1 patient (1.4%) receiving placebo (p value = 0.99). The overall rate of development of urticarial rash during the first 24 hours after RBC transfusion was 22% in the patients randomized to receive active drugs comparing with 35.2% in the patients receiving placebo as pretransfusion medications (p value = 0.111). The development of urticarial rash within 4 hours of RBC transfusion was noted in 8 (11%) and 7 (9.5%) patients randomized to receive active drugs and placebo, respectively (p value = 0.764). Interestingly, the delayed development of urticarial rash at 4 to 24 hours post RBC transfusion was reported in 19 patients (25.7%) randomized to placebo, which was statistically significantly higher than 8 patients (11%) receiving active drugs with a p value of 0.02 (OR = 0.35, 95%CI: 0.145-0.877,). There were no other transfusion reactions such as hemolytic transfusion reactions or other allergic reactions such as facial edema, airway edema, lower respiratory tract symptoms, and hypotension noted in all thalassemia patients enrolled in our study. Moreover, none of the patients developed adverse effects from IV CPM including drowsiness and thickening of bronchial secretions.

### 3.3. Risk Factors for Development of Urticarial Rash at 4 to 24 Hours Post-RBC Transfusion

Since the delayed development of urticarial rash at 4 to 24 hours post RBC transfusion was statistically significantly higher in thalassemia patents randomized to receive placebo comparing to those receiving active drugs consisting of oral acetaminophen and IV CPM as premedications prior to RBC transfusion, we further analyzed other associated risk factors which could potentially contribute to the delayed posttransfusion urticarial rash by using binary logistic regression analysis (Table 3). We found that types of thalassemia, type of RBC products, and patients' age were not associated with the development of posttransfusion urticarial rash at 4 to 24 hours (p-value > 0.05). However, patients' gender and premedications were noted to be risk factors for the development of post-transfusion urticarial rash. The development of delayed posttransfusion urticarial rash at 4 to 24 hours was statistically significantly higher in female (17 patients, 26.2%) than male (10 patients, 12.2%) with a p value of 0.03 (adjusted OR = 2.9, 95%CI: 1.204-7.426). Additional risk factor found to have statistical significant association with delayed posttransfusion urticarial rash was the use of IV CPM as premedication prior to RBC transfusion with a p-value of 0.025 (adjusted OR = 0.26, 95%CI: 0.101-0.689).

### 3.4. Risk Factors for Development of Febrile Reaction Post RBC Transfusion

We found that types of thalassemia, RBC products, patients' age, gender, and premedications were not associated with the development of febrile reaction (p value > 0.05) (Table 4).

## 4. Discussion

Thalassemia is a common inherited blood disorder characterized by decreased or absent hemoglobin production causing chronic hemolysis. The severity of the disease depends on the type of thalassemia ranging from a silent carrier with no clinical anemia and normal hemoglobin level to a severe case presenting with significant anemia requiring regular RBC transfusion. Although thalassemia disease can be cured by hematologic stem cell transplantation or gene therapy [26], financial limitation is still a major issue especially in developing countries where most of the patients ended up with chronic RBC transfusion. The side effects of RBC transfusion include iron overload requiring iron chelation and more importantly posttransfusion reactions with each cycle of RBC transfusion, which could potentially be fatal. The common transfusion reactions are febrile reaction and allergic reactions in which the patients could present with urticarial rash, facial edema, airway edema, lower respiratory tract symptoms or more severe symptoms such as hypotension and anaphylaxis. The methods to minimize or prevent the development of transfusion reactions are different among institutional practice guidelines ranging from close observation to the use of pharmacologic premedications prior to RBC transfusion. Herein, the aim of our study was to evaluate the role of using pretransfusion medication consisting of oral acetaminophen and IV CPM comparing to placebo specifically in children and adolescent with thalassemia disease.

Children and adolescent with thalassemia disease who required RBC transfusion were enrolled in our study and blindly randomized to receive active drugs consisting of oral acetaminophen and IV CPM versus placebo prior to RBC transfusion. Although there were no statistical differences in the overall development of febrile reaction and urticarial rash during 24 hours of RBC transfusion, we found that the development of delayed urticarial rash from 4 to 24 hours after RBC transfusion was statistically significantly higher in the patients randomized to receive placebo comparing to those receiving active drugs. Furthermore, binary logistic regression analysis was used to investigate potential associated risk factors that could potentially contribute to the delayed posttransfusion urticarial rash which confirm the requirement of pretransfusion antihistamine.

There are many types of antihistamine available in the market and some of those were used as premedication prior to RBC transfusion. Bennardello F et al. found that the regimen of using oral loratadine taken by the patients at home for 3 consecutive days prior to transfusion could reduce allergic reactions to 0.9% in patients receiving washing and/or double filtration of RBC products [27]. However, the use of antihistamine in oral form and to give the drug for several days prior to transfusion might not be perfectly applicable in children and adolescent due to their reliability to take oral medication and potentially poor compliance especially in adolescent. In our study, we decided to use IV CPM as premedicated antihistamine and administered this medication shortly prior to RBC transfusion in order to cut off those concerns. CPM is a H1-receptor antagonist, and it blocks a certain kind of histamine receptor (H1 receptor) required for the uptake of histamine among the cells leading to the prevention of allergic symptoms. Although Kennedy LD et al. used diphenhydramine which is the same first generation antihistamine as CPM and found no significant difference in the development of allergic reactions between patients receiving diphenhydramine and those receiving placebo, the study only looked at immediate reaction within 4 hours of transfusion [18]. According to the results from our study, there was no significant difference in the development of urticarial rash within 4 hours of RBC transfusion between patients randomized to receive IV CPM and those receiving placebo which is also resemble to the study from Kennedy LD et al. However, we further followed up patients up to 24 hours and noted that the incidence of delayed development of urticarial rash at 4 to 24 hours post-RBC transfusion was significantly lower in patients randomized to receive IV CPM than those receiving placebo. Our study affirms the role of IV CPM in preventing delayed development of urticarial rash which is important since during that time interval, the patients are at home where the close monitoring of transfusion reactions by healthcare professional is not feasible. This finding also confirms the need to monitor patients for a longer period of time instead of discharging patients immediately once the RBC transfusion is completed. In addition, we also found that delayed development of urticarial rash was significantly higher in female than in male. This interesting finding could be from the effect of hormone estrogen in female on the enzyme lining the blood vessels causing the increased production of nitric oxide which results in prolonged and severe allergic reactions [28, 29]. Moreover, a combination of CPM (H1-receptor antagonist) and ranitidine (H2-receptor antagonist) provided a satisfactory result in urticarial treatment [30]. Therefore, it might be interesting to evaluate this combination of H1- and H2-receptor antagonists as pretransfusion medication in order to prevent the development of urticarial rash in the future study.

Compared to incidence reported in literatures, the higher incidence of transfusion reactions in our study could be from our patient population's background. Since our study was conducted on thalassemia patients who required regular red cell transfusion every 3 to 4 weeks, frequent exposure to antigens from different red cell products might cause increased alloreactivity to antigens expressed on donor cells and be the reason for a higher risk of development of febrile reaction as well as allergic reactions. In addition, our study had monitored transfusion reactions for a longer period of time up to 24 hours after red cell transfusion using follow-up phone call and was able to detect delayed development of reactions. Therefore, this might be another reason for a higher incidence of reactions in our study.

The study done by KK Tan et al. reported the significant increase of transfusion reactions in patients receiving buffy coat-poor packed red cell products comparing to filtered blood products through leukocyte-filter (11.9% versus 2.6%, respectively) [31] with no statistical difference of febrile reaction between those two blood products. In Thailand, the standard transfused RBC products are LDPRC (prestorage leukoreduction) and LPRC (buffy coat-poor packed red cell prepared by centrifuge). In our study, we found no statistical difference of febrile reaction as well as urticarial rash between the patients receiving LDPRC and those receiving LPRC. This finding assures us to confidently transfuse LPRC to patients when LDPRC is not available, and the use of premedicated oral acetaminophen prior to transfusion might not be necessary in those patients.

Unlike other transfusion reactions papers, our study has focused on a specific type of patient population (pediatric and adolescent patient) with specific type of disease (thalassemia disease). Since thalassemia is the most common inherited blood disorder especially in South East Asia and Mediterranean and most of the patients affected with this disease unfortunately require regular blood transfusion throughout their life, many of them are suffered from transfusion complications. In addition, in our study, we extended the duration of observation and monitoring of transfusion reactions to 24 hours, and interestingly we found the higher incidence of delayed development of urticarial rash, which could be successfully decreased by administration of intravenous chlorpheniramine maleate. We truly hope that the data from our study could be used and beneficial for other practitioners in order to improve the ways we manage and monitor those patients in clinical practice.

In conclusion, this study is a prospective, randomized, double-blind placebo controlled trial. Based on the results of our study, we recommend that the use of oral acetaminophen as pretransfusion medication to prevent febrile reaction is not necessary in thalassemia patients who receive LPRC and LDPRC transfusion. However, IV CPM might be beneficial as premedication to prevent delayed urticarial rash in those patients especially in female. Furthermore, the patient should be informed to observe signs and symptoms of delayed development of allergic reactions and to contact healthcare professional if the symptoms occur.

## Figures and Tables

**Table 1:** Patients' demographic data.

	**Active drugs**	**Placebo**	**p-value**
	**(N=73)**	**(N=74)**	
**Gender**			
Male	37(50.7)	45(60.8)	0.216
Female	36(49.3)	29(39.2)	
**Types of Thalassemia**			
ß thal/ß thal	12(16.4)	7(9.5)	0.353
ß thal/Hb E	49(67.1)	50(67.5)	
AE Bart disease	6(8.2)	10(13.5)	
Hb H with CS	6(8.2)	7(9.5)	
**RBC products**			
LPRC	49(67.1)	50(67.6)	0.954
LDPRC	24(32.9)	24(32.4)	
**Weight (kg)**			
Mean±SD	37.7±15.81	33.45±15.07	0.138
Min-Max	10.3-62.3	10.7-61.5	
**Age (years)**			
Mean±SD	12.49±4.84	11.32±4.72	0.097
Min-Max	1.58-18.5	1.5-21.17	
**Age at diagnosis (months)**			
Mean±SD	24.93±23.84	25.08±24.36	0.970
Min-Max	0.0-87.0	0.0-122	

***Note:*** *Data were shown as number (%), mean±SD, min-max; p values were analyzed by Chi-square test for categorical data and independent sample t-test for continuous data. p<0.05 is statistically significant.*

***Abbreviations.*** *ß thal/ß thal: homozygous beta thalassemia; ß thal/HbE: beta thalassemia/hemoglobin E; Hb H with CS: hemoglobin H disease with constant spring; RBC: red blood cell; LPRC: leukocyte poor packed red cell; LDPRC: leukocyte depleted packed red cell.*

**Table 2:** The associated risk between premedications and transfusion reactions.

	**Active drugs**	**Placebo**	**p-value**	**OR**	**95%CI**
	**(N=73)**	**(N=74)**			
**Fever 0 - 4 hrs**					
Yes	4(5.5)	6(8.1)	0.527	1.522	0.411-5.634
No	69(94.5)	68(91.9)			
**Fever 4 - 24 hrs**					
Yes	1(1.4)	1(1.4)	0.992	1.014	0.062-16.521
No	72(98.6)	73(98.6)			
**Rash 0 - 4 hrs**					
Yes	8(11)	7(9.5)	0.764	1.178	0.404-3.435
No	65(89)	67(90.5)			
**Rash 4 - 24 hrs**					
Yes	8(11)	19(25.7)	**0.021** **∗**	0.356	0.145-0.877
No	65(89)	55(74.3)			

***Note.*** *Data were shown as number (%), and associated risks between treatment groups were analyzed by binary logistic regression;* **∗**: *p<0.05 is statistically significant.*

**Table 3:** Risk factors for development of urticarial rash at 4 to 24 hours post-RBC transfusion.

	**Rash 4 - 24 hrs**		**p-value**	**Crude OR**	**95%CI**	**Adjusted OR**	**95%CI**
	**Yes**	**No**					
	**N = 27**	**N = 120**					
**Thalassemia types**							
ß thal/ß thal	5(26.3)	14(73.7)	0.159	3.095	0.643-14.906		
ß thal/Hb E	19(19.2)	80(80.8)	0.275	2.058	0.563-7.519		
Others	3(10.3)	26(89.7)		1			
**RBC products**							
LPRC	19(19.2)	80(80.8)	0.711	1.187	0.478-2.947		
LDPRC	8(16.7)	40(83.3)					
**Age**							
	13.22±4.58	11.61±4.81	0.118	1.079	0.981-1.187	1.103	0.993-1.225
**Gender**							
Male	10(12.2)	72(87.8)		1		1	
Female	17(26.2)	48(73.8)	**0.033** **∗**	2.55	1.077-6.04	**2.990** **∗**	1.204-7.426
**Premedications**							
Active drugs	8(11)	65(89)	**0.025** **∗**	0.356	0.145-0.877	**0.263** **∗**	0.101-0.689
Placebo	19(25.7)	55(74.3)		1		1	

***Note.*** *Data were shown as number (%), and associated risks between treatment groups were analyzed by binary logistic regression;* **∗**: *p<0.05 is statistically significant.*

***Abbreviations.*** *ß thal/ß thal: homozygous beta thalassemia; ß thal/HbE: beta thalassemia/hemoglobin E; RBC: red blood cell; LPRC: leukocyte poor packed red cell; LDPRC: leukocyte depleted packed red cell.*

**Table 4:** Risk factors for development of febrile reaction post RBC transfusion.

	**Febrile reaction**		**p-value**	**Crude OR**	**95%CI**
	**Yes**	**No**			
	**N = 12**	**N = 135**			
**Thalassemia**					
ß thal/ß thal	3(15.8)	16(84.2)	0.166	5.250	0.503-54.777
ß thal/Hb E	8(8.1)	91(91.9)	0.405	2.462	0.295-20.540
Other	1(3.4)	28(96.6)		1	
**RBC products**					
LPRC	9(9.1)	90(90.9)	0.557	1.500	0.387-5.814
LDPRC	3(6.2)	45(93.8)		1	
**Age**					
	13.9±3.63	11.73±4.86	0.141	1.116	0.964-1.291
**Gender**					
Male	7(8.5)	75(91.5)		1	
Female	5(7.7)	60(92.3)	0.853	0.893	0.270-2.955
**Premedications**					
Active drugs	5(6.8)	68(93.2)	0.565	0.704	0.213-2.328
Placebo	7(9.5)	67(90.5)		1	

***Note.*** *Data were shown as number (%), mean±SD, and associated risks between treatment groups were analyzed by binary logistic regression; p<0.05 is statistically significant.*

***Abbreviations.*** *ß thal/ß thal: homozygous beta thalassemia; ß thal/HbE: beta thalassemia/hemoglobin E; RBC: red blood cell; LPRC: leukocyte poor packed red cell; LDPRC: leukocyte depleted packed red cell*

## Data Availability

The data used to support the findings of this study are available from the corresponding author upon request.
